# Identifying local foci of tuberculosis transmission in Moldova using a spatial multinomial logistic regression model

**DOI:** 10.1016/j.ebiom.2024.105085

**Published:** 2024-03-26

**Authors:** Yu Lan, Valeriu Crudu, Nelly Ciobanu, Alexandru Codreanu, Melanie H. Chitwood, Benjamin Sobkowiak, Joshua L. Warren, Ted Cohen

**Affiliations:** a Department of Epidemiology of Microbial Diseases, Yale School of Public Health, New Haven, CT, USA; b Department of Biostatistics, Yale School of Public Health, New Haven, CT, USA; c Phthisiopneumology Institute, Chisinau, Republic of Moldova

**Keywords:** Tuberculosis, MDR-tuberculosis, Transmission foci, Hierarchical bayesian methods, Spatial statistics

## Abstract

**Background:**

Multidrug resistant tuberculosis (MDR-TB) represents a major public health concern in the Republic of Moldova, with an estimated 31% of new and 56% of previously treated TB cases having MDR disease in 2022. A recent genomic epidemiology study of incident TB occurring in 2018 and 2019 found that 92% of MDR-TB was the result of transmission. The MDR phenotype was concentrated among two *M. tuberculosis* (*Mtb*) lineages: L2.2.1 (Beijing) and L4.2.1 (Ural).

**Methods:**

We developed and applied a hierarchical Bayesian multinominal logistic regression model to *Mtb* genomic, spatial, and epidemiological data collected from all individuals with diagnosed TB in Moldova in 2018 and 2019 to identify locations in which specific *Mtb* strains are being transmitted. We then used a logistic regression model to estimate locality-level factors associated with local transmission.

**Findings:**

We found differences in the spatial distribution and degree of local concentration of disease due to specific strains of Beijing and Ural lineage *Mtb*. Foci of transmission for four strains of Beijing lineage *Mtb*, predominantly of the MDR-TB phenotype, were located in several regions, but largely concentrated in Transnistria. In contrast, transmission of Ural lineage *Mtb* had less marked patterns of spatial aggregation, with a single strain (also of the MDR phenotype) spatially clustered in southern Transnistria. We found a 30% (95% credible interval 2%–80%) increase in odds of a locality being a transmission cluster for each increase of 100 persons per square kilometer, while higher local tuberculosis incidence and poverty were not associated with a locality being a transmission focus.

**Interpretation:**

Our results identified localities where specific *Mtb* transmission networks were concentrated and quantified the association between locality-level factors and focal transmission. This analysis revealed Transnistria as the primary area where specific *Mtb* strains (predominantly of the MDR-TB phenotype) were locally transmitted and suggests that targeted intensified case finding in this region may be an attractive policy option.

**Funding:**

Funding for this work was provided by the 10.13039/100000060National Institute of Allergy and Infectious Diseases at the US National Institutes of Health.

**Research in context**

**Evidence before this study**

We searched PubMed using the terms (genomic OR genome OR sequencing) AND (spatial analysis OR spatial OR geospatial) AND (tuberculosis OR TB) to identify primary research studies on integrating genomic analysis and spatial analysis to locate tuberculosis (TB) transmission foci. Several previous studies have reported that the transmission of strains of *M. tuberculosis* may be geographically restricted, but areas with high incidence rates are not always areas in which transmission is focused. Previous transmission dynamic modeling has suggested that interventions targeted to transmission foci may be an attractive approach for improving TB control, but there is limited evidence to demonstrate the effectiveness of targeted approaches, especially for control of multidrug resistant TB (MDR-TB).

**Added value of this study**

We developed a two-step approach to use pathogen genomic and spatial data to i) identify villages in which specific strains of *M. tuberculosis* are concentrated and ii) identify village-level factors associated with local transmission. We apply this approach to a prospective study in the Republic of Moldova, where greater than 90% of MDR-TB occurs through transmission. We found differences in the spatial distribution and degree of local concentration of TB due to specific strains of Beijing and Ural lineage *M. tuberculosis*, the two lineages in which MDR is concentrated in Moldova and that villages with higher population densities were more likely to be transmission hotspots. We also found that simple rules for targeting interventions (e.g., based on TB notification rates) may often direct targeted interventions to areas that are not foci of local transmission.

**Implications of all the available evidence**

Our findings highlight the role of pathogen genomic data for understanding the local transmission patterns of TB and MDR-TB in high incidence settings like the Republic of Moldova. We found distinct patterns of spatial distribution of the two major lineages responsible for MDR-TB in Moldova, which may reflect differences in the evolutionary history and reproductive potential of highly drug resistant *M. tuberculosis*. Further work is needed to understand the role that pathogen genomic data can play in the planning of public health interventions, such as targeting active case finding to areas in which transmission is most intense.

## Introduction

Globally, more than 400,000 individuals developed multidrug-resistant tuberculosis (MDR-TB) in 2021.^1^ MDR-TB, defined as TB resistant to at least isoniazid and rifampin, is a leading cause of antimicrobial resistance-associated mortality.^2^ It has recently been estimated that greater than 95% of all incident MDR-TB now results from direct transmission^3^ (as opposed to resistance acquisition through selection during treatment of drug susceptible TB), thus efforts to reduce the incidence of MDR-TB must rely on interventions that can stop MDR-TB transmission.

Interventions that interrupt MDR-TB transmission depend on prompt detection and diagnosis and effective treatment of individuals with MDR-TB. Over the past decade, major strides have been made in the development of reliable point-of-care tools for detecting drug resistance at the time of diagnosis (e.g., Xpert MDR-TB which simultaneously identifies TB and rifampin resistance^4^) as well as in the development of shorter, more effective, and less toxic MDR-TB regimens (e.g., BPaL^5^). Despite this progress, as of 2021, fewer than 50% of estimated individuals with incident MDR-TB received diagnosis and appropriate treatment. This grim statistic underscores the urgency of new approaches to improve access to diagnosis and care of MDR-TB.

Reductions in costs associated with genomic sequencing and the collection of detailed spatial data have accelerated the application of phylogeographic analyses for understanding the transmission of pathogens, including *M. tuberculosis* (*Mtb*). Use of these methods has revealed that the transmission of strains of *Mtb* may be geographically restricted,^6^^,^^7^ even to within a single city, and that local heterogeneity in TB notification rates may be a poor proxy for these transmission foci,^8^ emphasizing the need for sequencing to identify transmission patterns. These local patterns of transmission also suggest that there may be opportunities for intensifying active case finding approaches in areas where most MDR-TB transmission occurs. Previous transmission dynamic modeling has suggested that interventions targeted to transmission foci may be an attractive tactic for improving TB control,^9^ but, to date, there is limited evidence to demonstrate the effectiveness of targeted approaches.^10^

Here, we aim to investigate the patterns of local transmission of TB and MDR-TB in the Republic of Moldova, a country where a large proportion of new cases (31%) and previously treated (56%) TB cases had rifampin-resistant or MDR-TB in 2021. We develop and apply a hierarchical Bayesian multinominal logistic regression model to *Mtb* genomic, spatial, and epidemiological data collected from all individuals with diagnosed TB in Moldova in 2018 and 2019 to identify localities in which specific *Mtb* strains are being transmitted and use logistic regression to estimate locality-level factors associated with local transmission.

## Methods

### Data and study setting

This analysis used data collected in a prospective observational study fully described in a previous publication.^11^ Briefly, we attempted to enroll and collect *Mtb* isolates and baseline demographic and clinical data from all culture-positive TB cases in the Republic of Moldova between January 1, 2018 and December 31, 2019.

In total, there were 2236 individuals included in the study, 36% of whom were infected with strains with the MDR phenotype. Phylogenetic analysis showed that MDR was concentrated within two *Mtb* lineages: L2.2.1 (Beijing) (n = 901 isolates, 48% of which were MDR) and L4.2.1 (Ural) (n = 535 isolates, 61% of which were MDR). We estimated that 92% of all MDR-TB in Moldova was found within putative transmission networks.

Previously,^11^ we used a phylogenetic clustering approach to identify thirty-five putative TB transmission networks comprising at least ten cases.^12^ Sixteen of these transmission networks involved Beijing strains, eighteen were Ural transmission networks, and there was one transmission network of *M. bovis*. Here, we seek to identify areas in which these large transmission networks were spatially focused, and to determine whether there are factors associated with a locality being an area of focal transmission of a particular strain of *Mtb*.

**Fig. 1:**
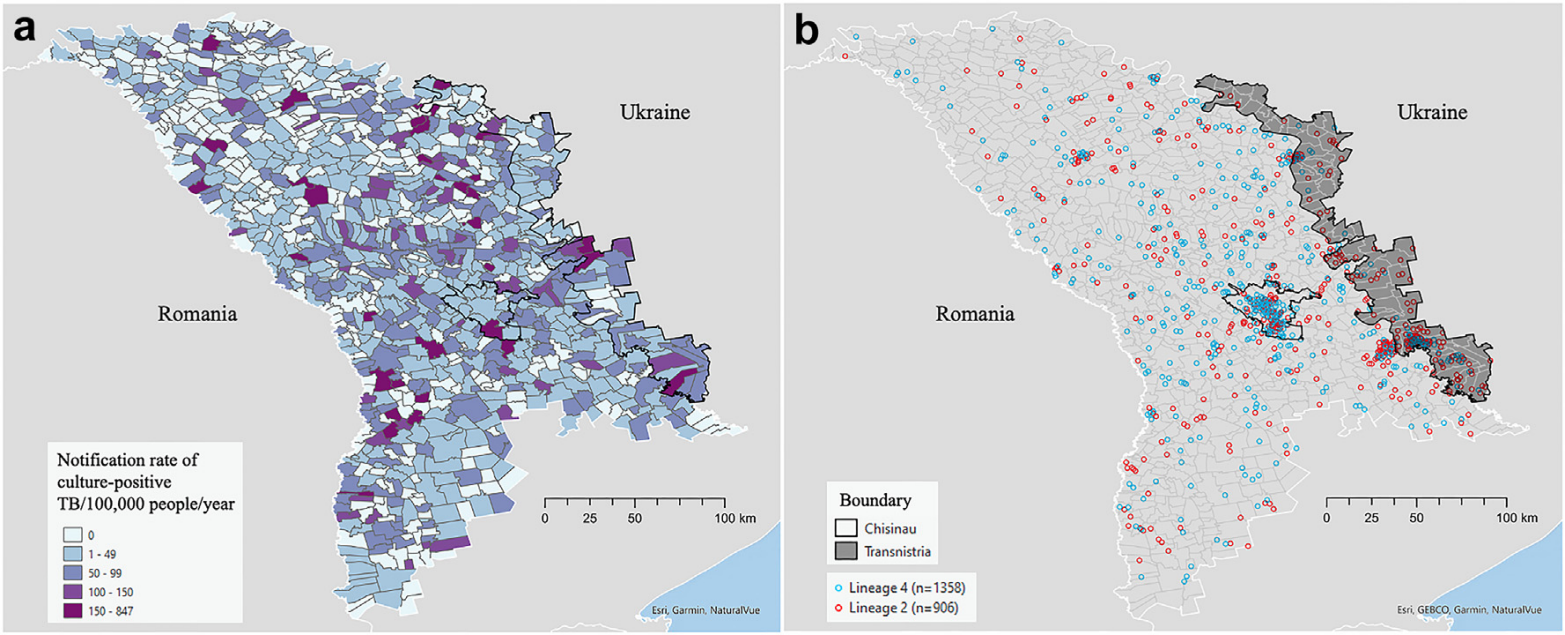
**Spatial distribution of culture-positive *Mtb* in Moldova. a) Notification rate of culture-positive TB/100,000/year for 2018–2019 at the locality level; b) Locality-level distribution of *Mtb* isolates by lineage. Locations have been jittered**.

Moldova is divided into 973 localities^13^; each individual with TB included in the study was assigned to their locality of residence at the time of diagnosis. Fig. 1 shows the locality-level notification rate of culture-positive TB in 2018–2019 and the spatial distribution of culture-positive *Mtb* by two major lineages.

We obtained population data for most localities from a 2014 census (the most recent national census); however, for localities located in Transnistria, a breakaway region along the eastern border of the country, we extracted population data from an alternative source^14^ as the data were not available from the census. As no poverty information is available at such level, we used a measure of the relative wealth index^15^ to represent the poverty status across the country. This public dataset includes the distribution of estimated relative poverty and wealth across 135 low- and middle-income countries by applying machine-learning algorithms to multiple data sources (e.g., satellites and mobile phone networks). We have provided maps of the population density and the relative wealth index in the Appendix (Figure S1).

### Statistics

#### Stage 1 model: identifying local foci of transmission

In the first analysis stage, we use a hierarchical Bayesian multinomial logistic regression framework to identify localities in which strains from a single transmission network are overrepresented. The model includes random effects, which are defined using a multivariate version of the spatial model from Besag, York, and Mollié (BYM)^16^ to simultaneously account for potential spatial correlation in the data and allow us to identify localities with excess risk (i.e., hotspots). For each locality, the vector of outcomes includes the total number of TB cases from each of the 35 large putative TB transmission networks with an extra control category when the case does not belong to any transmission network. This vector is modeled as

(1) $$Y_{i}|p_{i}∼\text{Multinomial}\left(N_{i};p_{i}\right),i=1,\ldots ,n,p_{ij}=\frac{\exp\left\{\beta_{j}\;+\;\theta_{ij}\;+\;\psi_{ij}\right\}}{\sum_{k=0}^{m}\exp\left\{\beta_{k}\;+\;\theta_{ik}\;+\;\psi_{ik}\right\}},j=0,\ldots ,m\text{,}$$

where $Y_{i}={\left(Y_{i0},\ldots ,Y_{im}\right)}^{T}$ is the vector of counts from locality *i* (*n* total localities) and $Y_{ij}$ is the total number of cases that belong to transmission network *j* (*m* total networks), with $Y_{i0}$ the number of non-networked cases; $N_{i}$ is the total number of TB cases in locality *i* (i.e., $\sum_{j=0}^{m}Y_{ij}=N_{i}$); $p_{i}={\left({p_{i0},\ldots ,p}_{im}\right)}^{T}$ is the vector of probabilities from locality *i* that define the expected number of TB cases in each category where $\sum_{j=0}^{m}p_{ij}=1$ for all localities; $\beta_{j}$ is the intercept parameter for category *j* shared across all localities; $\theta_{ij}$ is a non-spatially correlated random effect specific to locality *i* and category *j* that accounts for correlation between random effects from the same locality but is independent across localities. Similarly, $\psi_{ij}$ is a spatially correlated random effect that follows the multivariate version of the intrinsic conditional autoregressive model^17^ which accounts for correlation between effects from the same locality and spatial correlation across localities. By including spatial and non-spatial random effects in the framework, the BYM model attempts to avoid spatial over-smoothing by using the data to estimate what proportion of the total variation is spatially vs. non-spatially structured.

Specifically, the vector of spatially correlated random from locality *i* (i.e., $\psi_{i}={\left(\psi_{i0},\ldots ,\psi_{im}\right)}^{T}$) is modeled as

(2) $$\psi_{i}|\psi_{-i},\tau^{2},\rho ∼\text{MVN}\left(\frac{\sum_{k=1}^{n}w_{ik}\psi_{k}}{\sum_{k=1}^{n}w_{ik}},\frac{\tau^{2}\Omega \left(\rho \right)}{\sum_{k=1}^{n}w_{ik}}\right),i=1,\ldots ,n\text{,}$$

where $\psi_{-i}$ is the set of all locality-specific vectors with the vector from the *i*th locality removed; $\tau^{2}$ is the variance parameter specific to the spatial random effects; Ω(ρ) is the m+1 by m+1 cross-covariance matrix that describes the correlation between parameters from the same locality where $\Omega {\left(\rho \right)}_{ij}=\rho$ for i≠j, Ω(ρ)=1 for i=j, and ρ∈(0,1) describes the strength of correlation between the parameters; and $w_{ik}$ are binary variables that are equal to one when localities *i* and *k* share a common border, and are equal to zero otherwise ($w_{ii}=0$ for all *i* by definition). A priori, this model suggests that random effect values from a specified locality are similar to a weighted average of values from neighboring localities.

The non-spatial random effects are modeled as $\theta_{i}∼\text{MVN}\left(0_{m+1},\sigma^{2}\Omega \left(\rho \right)\right),i=1,\ldots ,n\text{,}$ where $\theta_{i}$ is ordered in the same was as $\psi_{i}$; $0_{m+1}$ is a vector with m+1 entries that are all equal to zero; and $\sigma^{2}$ is the non-spatial variance parameter.

To complete the model specification, we assigned weakly informative prior distributions for the introduced model parameters such that $\beta_{j}∼N\left(0,\delta^{2}\right),j=1,\ldots ,m$; $\delta^{2},\tau^{2},\sigma^{2}∼\text{Inverse}\;\text{Gamma}\left(0.01,0.01\right)$; and $\ln\left(\frac{\rho }{1-\rho }\right)∼N\left({0,100}^{2}\right)$.

To identify localities in which strains from a single transmission network were overrepresented, we make inference on the $\theta_{ij}\;+\;\psi_{ij}$ random effects. Specifically, if strain *j* was equally represented across all localities, we would expect $\theta_{ij}\;+\;\psi_{ij}=0$ for all *i*. However, if this strain is overrepresented in locality *i*, we expect $\theta_{ij}+\psi_{ij}>0$. Therefore, after fitting the model, we identify $\theta_{ij}\;+\;\psi_{ij}$ parameters whose 95% credible intervals are entirely above zero and classify these areas as transmission foci if any of the strain-specific effects meet this criterion.

Accounting for spatial correlation in these effects provides regularization so that the results are less sensitive to outlying values and results are more robust. Importantly, we do not specify the level of spatial correlation in the model and allow the data to determine the appropriate amount (i.e., through estimation of $\tau^{2}$ and $\sigma^{2}$).

To test the robustness of this approach for identifying putative transmission foci, we also applied a non-parametric distance-based method previously used to identify TB transmission foci based on genotyping data^6^ (Appendix).

#### Stage 2 model: identifying locality-level factors with being a focus of Mtb transmission

In a second stage analysis, we used a hierarchical Bayesian logistic regression modeling framework to identify locality-level factors associated with being flagged as a locus of transmission of a specific strain in the first stage modeling. This model also included spatially correlated random effects which followed the standard intrinsic conditional autoregressive model. Specifically, we define $Z_{i}=1$ if locality *i* was identified as a focus (for any of the strains) in the first stage analysis and model the probability of this outcome (i.e., $\pi_{i}$) as

(3) $$\text{logit}\left(\pi_{i}\right)=x_{i}^{T}\gamma \;+\;\phi_{i}$$

where $x_{i}$ is a vector of predictors specific to locality *i* and $\phi_{i}$ is the spatial random effect which serves to account for spatial correlation in the outcome, leading to accurate statistical inference for the primary regression parameters of interest (i.e., γ). These random effects have a similar prior model as the $\psi_{ij}$ parameters from equation (2), but are univariate at each locality instead of multivariate, with $\tau^{2}\Omega \left(\rho \right)$ replaced by the scalar variance parameter $\tau^{2}$. We assigned weakly informative prior distributions for the introduced model parameters such that $\gamma_{j}∼N\left(0,100^{2}\right)$ and $\tau^{2}∼\text{Inverse}\;\text{Gamma}\left(0.01,0.01\right)$. These locality-level factors included population density, relative wealth index, and culture-positive TB notification rate.

We used the Integrated Nested Laplace Approximation (INLA) software^18^^,^^19^ in R for model fitting in both stages. INLA provides a computationally tractable solution for fitting high dimensional Bayesian methods (e.g., spatial models) to large datasets by accurately approximating the marginal posterior distributions of the individual model parameters. For fitting the first stage multinomial logistic regression model in INLA, we relied on the Multinomial-Poisson transformation with sum-to-zero constraints on the intercepts and random effects as described elsewhere^20^ as INLA is currently unable to directly model multinomially distributed outcomes.

For both analyses (Stages 1 and 2), we reran the models with alternative weakly informative prior distributions to assess the sensitivity of the findings to these priors. Specifically, in the first stage the 0.01 values used in the inverse gamma priors for $\tau^{2}$ and $\sigma^{2}$ were replaced by 0.10, and the standard deviation of 100 used in the Gaussian distribution for $\ln\left(\frac{\rho }{1-\rho }\right)$ was replaced by 10 (see Figure S2). Similarly, in the second stage 0.10 was used for the inverse gamma prior distribution and 10 was used as the standard deviation of the fixed effect regression parameters.

### Role of funders

This Study was funded by the US NIH. The funder had no role in the collection, analysis or interpretation of data and no involvement in writing or the decision to submit for publication. Authors of the study were not precluded from accessing data included in the study and accept responsibility to submit for publication.

### Ethics

This study was approved by the Ethics Committee of Research of the Phthisiopneumology Institute in Moldova and the Yale University Human Investigation Committee (No. 2000023071). Written informed consent of participants was obtained.

## Results

### Transmission foci

**Fig. 2:**
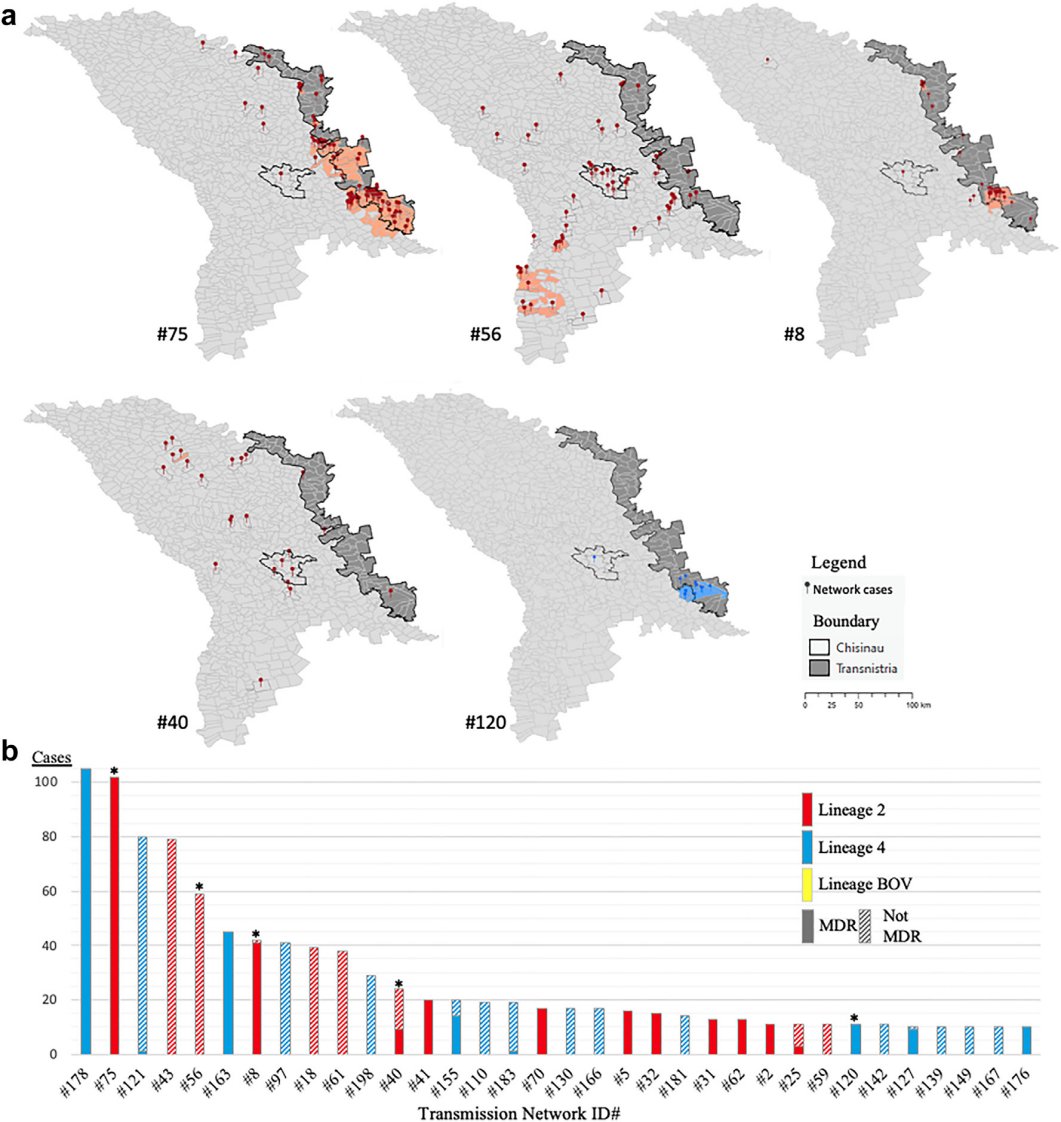
S**patial distribution and sizes of transmission networks. a) Localities in which specific strains (i.e., transmission networks) of *M. tuberculosis* is focused (red markers represent Beijing lineage cases and salmon localities show foci of local transmission of these strains, blue markers represent Ural lineage cases and light blue localities show local foci of transmission of this strain); b) Putative TB transmission networks by size, lineage, and fraction which are MDR. Blue colored bars represent Ural lineage transmission networks and red colored bars represent Beijing lineage transmission networks. The yellow bar is a single putative transmission network of *M. bovis*. The fraction of each bar which is solid color represents the fraction of that transmission network that has the MDR phenotype. Only transmission networks with at least 10 cases are included here (n = 35). ∗indicates the a network was found to have at least one focus of transmission (mapped in panel A)**.

We identified a total of 63 localities where transmission of specific *Mtb* strains was concentrated. 57 localities were foci of Beijing lineage strain transmission while only 6 localities were foci of Ural lineage strain transmission, and 5 localities were transmission foci of strains of both Beijing and Ural lineages (Fig. 2a). Only 5 of the 35 large transmission networks had evidence of focal transmission in one or more localities (Fig. 2b).

**Fig. 3:**
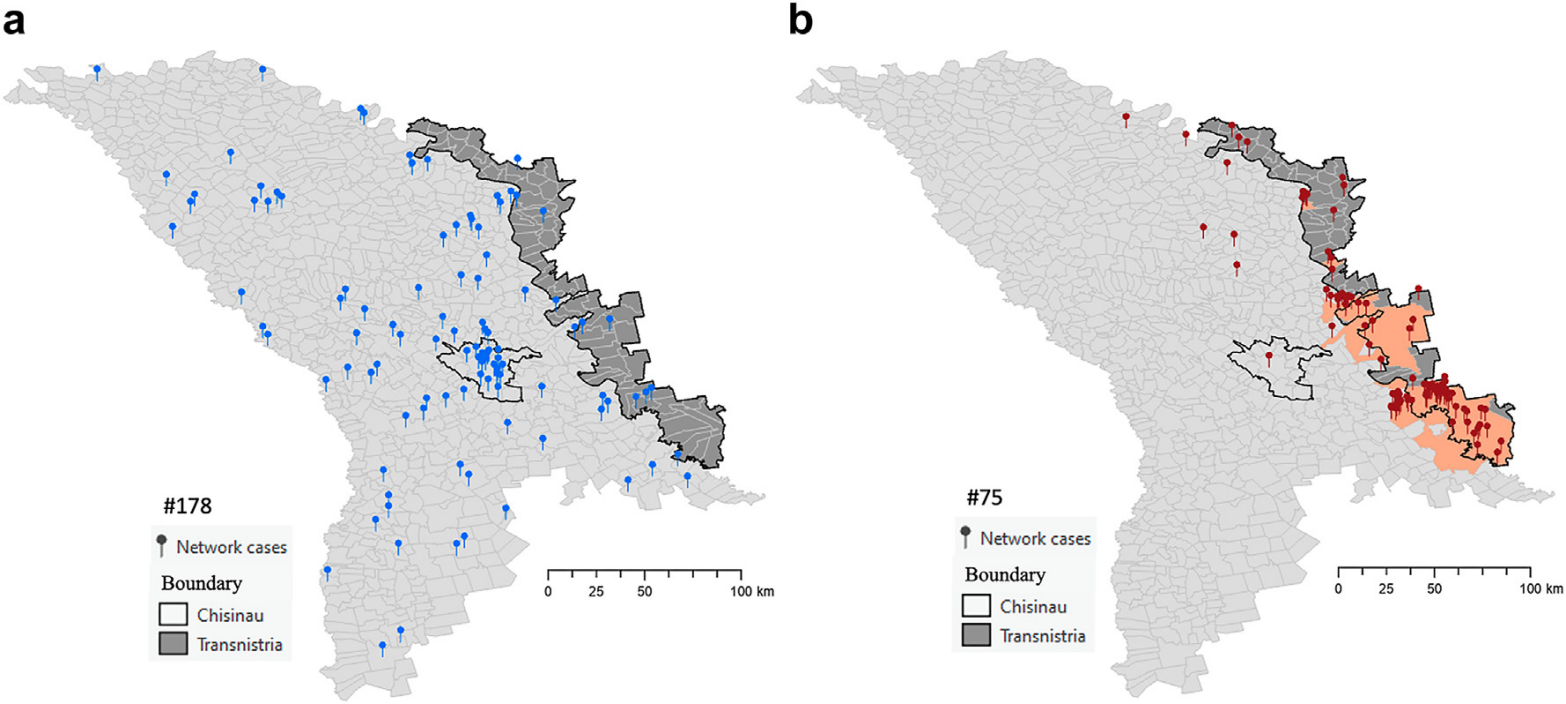
**Comparing the spatial distribution of cases from two largest transmission networks: a) Network 178 (n = 105 individuals) of the largest Ural strain (Lineage 4) shows no areas of focal transmission; b) Network 75 (n = 102 individuals) of the largest Beijing strain Lineage 2 is predominantly concentrated in localities detected as transmission foci**.

Three of four largest Beijing lineage transmission networks (ID#s 75, 56, 8) had locality-level transmission foci. The largest Beijing transmission network (#75, n = 102 individuals) was comprised entirely of the MDR phenotype and most of cases (84.3%) were found in localities identified as transmission hotspots (Figs. 2a and 3b). A second relatively large Beijing transmission network dominated with strains of the MDR phenotype (#8, n = 42 individuals) was also largely (73.8%) found in transmission hotspots. We also note the presence of a Beijing lineage transmission network comprised of strains that were not MDR with geographic focus (e.g., #56, with 23.7% of member isolates located in transmission foci).

In contrast, the two largest Ural lineage transmission networks (ID#s 178 and 121) did not have any locality-level transmission foci (Fig. 2b). The largest Ural transmission network (#178, n = 105 individuals) was also comprised entirely of the MDR phenotype and was widely distributed across the country without any areas of focused transmission (Fig. 3a). Only one Ural transmission network (#120, n = 11) with entirely MDR phenotype was predominantly (72.7%) found in transmission foci.

While both Beijing and Ural lineage transmission networks were widely distributed across Moldova (Fig. 1b), the Transnistria region was the area where local transmission of specific *Mtb* strains of MDR phenotype (i.e., ID#s 75, 8, 120) was most concentrated (Fig. 2). In particular, throughout Transnistria we identified localities in which the largest Beijing lineage strain (network #75) was being transmitted and in the southern portion of Transnistria we identified localities in which one Beijing lineage strain (network #8) and one Ural lineage strain (network #120) were also concentrated.

Comparison of localities identified as transmission hotspots with the hierarchical Bayesian multinomial logistic regression model were broadly similar to those identified with the non-parametric distance-based method (Appendix), with areas of primary concentration of local transmission in Transnistria.

### Locality-level factors with local transmission

The odds (posterior mean) of a locality being a hotspot for local transmission was increased by 30% (quantile-based 95% credible intervals [CrIs]: 2%–80%) for each increase of 100 persons per square kilometer. The culture positive TB notification rates (posterior mean odds ratio = 1.04; 95% CrIs 0.94–1.16) and poverty (i.e., the relative wealth index, posterior mean odds ratio = 1.00; 95% CrIs 0.01–6.32) predictors were not statistically significantly associated with a locality being a focus of local transmission.

**Fig. 4:**
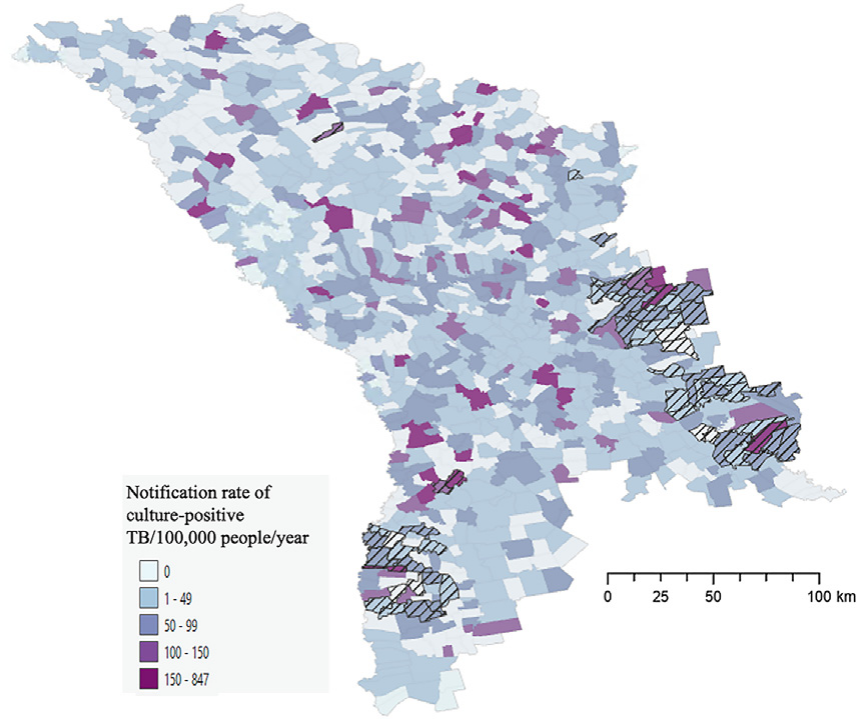
**Localities with high TB notification rates are not necessarily areas where focal transmission was detected. Notification rate of culture-positive TB/100,000/year for 2018–2019 overlayed with highlighted transmission foci (hatch filled) at the locality level demonstrating that many localities with high notification rates were not areas identified as foci of local transmission**.

Our result suggests that using notification rate alone as a predictor of locality-level transmission focus would be suboptimal. Although some localities with large notification rates were identified as transmission foci, many other localities with high notification rates were not foci of local transmission (Fig. 4).

## Discussion

In this analysis, we found differences in the spatial distribution and degree of local concentration of disease due to specific strains of Beijing and Ural lineage *Mtb*, the two lineages in which MDR is concentrated in Moldova. Foci of transmission of several strains of Beijing lineage *Mtb*, predominantly of the MDR-TB phenotype, were located throughout Transnistria. Our finding that Beijing lineage *Mtb* is an important driver of localized MDR transmission in Moldova is consistent with the epidemiological success of Beijing lineage strains that has been described in a number of previous studies.^21^, ^22^, ^23^, ^24^

In contrast to Beijing lineage strains, transmission networks of Ural lineage strains appeared to have less marked patterns of local aggregation, especially for those with an MDR phenotype. For example, the two largest transmission networks involving Ural strains (#178, n = 105, and #163, n = 45) all of which were of the MDR phenotype, were not concentrated in any particular localities. The wide geographic distribution of these large MDR TB transmission networks suggests that these two strains may have been spreading for many years within Moldova prior to the sampling frame for this study. While several previous studies have reported on the presence of MDR among small numbers of *Mtb* Ural strains in Moldova,^25^^,^^26^ the wide geographic spread of these strains suggests that MDR strains of the Ural lineage may have also spread more widely in the eastern European region than is currently recognized.

We found that increasing population density was associated with an increased odds of a locality being a focus of transmission. TB notification rate and local poverty were not statistically significantly associated with local transmission of strains. Simple decision rules, such as those based on locality-level TB notification rates, may be attractive options for prioritizing the use of intensified case finding. However, these types of heuristics would lead to substantial case finding efforts being focused on areas that may not be locations where local transmission was occurring. Accordingly, this analysis demonstrates the unique role of pathogen genomic data for pinpointing local areas of TB transmission. Future work will need to address the value of genomic surveillance for informing public health responses; at this time, it remains unclear whether and how genomic data can be gathered to inform timely interventions and if these genomic approaches offer sufficient benefits to justify their costs.

Our country-wide analysis of transmission included near all culture-positive TB cases identified in the Moldova over a two-year period. However, TB epidemics unfold over decades, and this short time window provides only a snapshot of ongoing transmission. Further, a substantial fraction of individuals with TB are diagnosed without microbiological confirmation, and the absence of a bacterial isolate precludes their inclusion in this type of genomic analysis. Our approach for inferring transmission networks addresses these issues,^27^ but these features of TB epidemics nonetheless challenge accurate reconstruction of TB transmission chains from observational data.

We applied a hierarchical Bayesian spatial multinomial logistic regression approach for our analysis. There are several benefits to this approach. First, this approach provides an automated, model-based and intuitive method for defining a transmission focus. That is, it allows us to identify localities in which a strain from a particular transmission network is overrepresented. This supports previous work suggesting that locations with significant spatial random effects can be used to detect areas of increased TB transmission^28^ and TB spillover from institutional settings.^29^ Second, it allows for us to make inference on meaningful spatial units (here we used localities, but this could be easily modified), which also allowed us to also explore the relationship between locality-level covariates and the locations of transmission foci. Finally, in comparison to approaches like DBM^30^ and kernel density estimation (KDE),^31^ the use of this regression approach avoids the need for arbitrary decisions about grid-size and bandwidth.

In conclusion, we found that analysis of spatial and genomic data revealed the location of localities where specific *Mtb* transmission networks were most concentrated and allowed us to quantify the relationship between increased population density and odds that a locality was flagged as a hotspot of local transmission. Our analyses revealed Transnistria as the primary area where transmission networks were focused in Moldova. This suggests the possibility that targeted intensified case finding in this regions may be an attractive policy option, but further modeling work is needed to estimate the potential epidemiological benefits and costs of this type of targeted screening.^10^

## Contributors

Conceptualization: TC, VC. Data curation: AC, NC, MHC. Formal analysis: YL, JLW, BS. Funding acquisition: TC, VC. Resources: TC. Supervision: TC, VC, JLW. Visualization: YL. Writing original draft: YL, TC, JLW. Writing review and editing: YL, VC, NC, AC, MHC, BS, JLW, TC. All authors read and approved the final manuscript.

## Data sharing statement

The genomic data have been made available through GenBank (PRJNA736718, https://www.ncbi.nlm.nih.gov/bioproject/PRJNA736718). Additional data used in the analysis (with the exception of location data which cannot be provided because of the small number of participants at locations), are provided as a csv in the Supporting information of reference 11 of this paper: Yang C, Sobkowiak B, Naidu V, Codreanu A, Ciobanu N, Gunasekera KS et al. Phylogeography and transmission of M. tuberculosis in Moldova: A prospective genomic analysis. PLOS Medicine. 2022;19 (2):e1003933.

## Declaration of interests

All authors do not have conflicting interests to declare.

## References

[1] World Health Organization Global tuberculosis report 2022. https://www.who.int/publications/i/item/9789240061729.

[2] Murray C.J., Ikuta K.S., Sharara F. (2022). Global burden of bacterial antimicrobial resistance in 2019: a systematic analysis. Lancet.

[3] Kendall E.A., Fofana M.O., Dowdy D.W. (2015). Burden of transmitted multidrug resistance in epidemics of tuberculosis: a transmission modelling analysis. Lancet Respir Med.

[4] Boehme C.C., Nabeta P., Hillemann D. (2010). Rapid molecular detection of tuberculosis and rifampin resistance. N Engl J Med.

[5] Nyang’wa B.-T., Berry C., Kazounis E. (2022). A 24-week, all-oral regimen for rifampin-resistant tuberculosis. N Engl J Med.

[6] Zelner J.L., Murray M.B., Becerra M.C. (2015). Identifying hotspots of multidrug-resistant tuberculosis transmission using spatial and molecular genetic data. J Infect Dis.

[7] Ribeiro F.K.C., Pan W., Bertolde A. (2015). Genotypic and spatial analysis of Mycobacterium tuberculosis transmission in a high-incidence urban setting. Clin Infect Dis.

[8] Huang C.-C., Trevisi L., Becerra M.C. (2022). Spatial scale of tuberculosis transmission in Lima, Peru. Proc Natl Acad Sci U S A.

[9] Dowdy D.W., Golub J.E., Chaisson R.E., Saraceni V. (2012). Heterogeneity in tuberculosis transmission and the role of geographic hotspots in propagating epidemics. Proc Natl Acad Sci U S A.

[10] Cudahy P.G.T., Andrews J.R., Bilinski A. (2019). Spatially targeted screening to reduce tuberculosis transmission in high-incidence settings. Lancet Infect Dis.

[11] Yang C., Sobkowiak B., Naidu V. (2022). Phylogeography and transmission of M. tuberculosis in Moldova: a prospective genomic analysis. PLoS Med.

[12] Balaban M., Moshiri N., Mai U., Jia X., Mirarab S. (2019). TreeCluster: clustering biological sequences using phylogenetic trees. PLoS One.

[13] ISCGM (2006). https://hgl.harvard.edu/catalog/sde-columbia-iscgm_moldova_2006_polbndl.

[14] Corbane C.F., Aneta, Pesaresi M., Politis P., Syrris V. (2018).

[15] Chi G., Fang H., Chatterjee S., Blumenstock J.E. (2022). Microestimates of wealth for all low- and middle-income countries. Proc Natl Acad Sci U S A.

[16] Besag J., York J., Mollié A. (1991). Bayesian image restoration, with two applications in spatial statistics. Ann Inst Stat Math.

[17] Gelfand A.E., Vounatsou P. (2003). Proper multivariate conditional autoregressive models for spatial data analysis. Biostatistics.

[18] Rue H., Martino S., Chopin N. (2009). Approximate Bayesian inference for latent Gaussian models by using integrated nested Laplace approximations. J Roy Stat Soc B Stat Methodol.

[19] Martins T.G., Simpson D., Lindgren F., Rue H. (2013). Bayesian computing with INLA: new features. Comput Stat Data Anal.

[20] Barmoudeh L., Baghishani H., Martino S. (2022). Bayesian spatial analysis of crash severity data with the INLA approach: assessment of different identification constraints. Accid Anal Prev.

[21] Merker M., Blin C., Mona S. (2015). Evolutionary history and global spread of the Mycobacterium tuberculosis Beijing lineage. Nat Genet.

[22] Casali N., Nikolayevskyy V., Balabanova Y. (2014). Evolution and transmission of drug-resistant tuberculosis in a Russian population. Nat Genet.

[23] Holt K.E., McAdam P., Thai P.V.K. (2018). Frequent transmission of the Mycobacterium tuberculosis Beijing lineage and positive selection for the EsxW Beijing variant in Vietnam. Nat Genet.

[24] Loiseau C., Windels E.M., Gygli S.M. (2023). The relative transmission fitness of multidrug-resistant Mycobacterium tuberculosis in a drug resistance hotspot. Nat Commun.

[25] Sinkov V., Ogarkov O., Mokrousov I., Bukin Y., Zhdanova S., Heysell S.K. (2018). New epidemic cluster of pre-extensively drug resistant isolates of Mycobacterium tuberculosis Ural family emerging in Eastern Europe. BMC Genom.

[26] Brown T.S., Eldholm V., Brynildsrud O. (2021). Evolution and emergence of multidrug-resistant Mycobacterium tuberculosis in Chisinau, Moldova. Microb Genom.

[27] Didelot X., Fraser C., Gardy J., Colijn C. (2017). Genomic infectious disease epidemiology in partially sampled and ongoing outbreaks. Mol Biol Evol.

[28] Gunasekera K.S., Zelner J., Becerra M.C. (2020). Children as sentinels of tuberculosis transmission: disease mapping of programmatic data. BMC Med.

[29] Warren J.L., Grandjean L., Moore D.A. (2018). Investigating spillover of multidrug-resistant tuberculosis from a prison: a spatial and molecular epidemiological analysis. BMC Med.

[30] Jeffery C., Ozonoff A., White L.F., Pagano M. (2013). Distance-based mapping of disease risk. Int J Biostat.

[31] Parzen E. (1962). On estimation of a probability density function and mode. Ann Math Stat.

