# Supplementary material for: Identifying local foci of tuberculosis transmission in Moldova using a spatial multinomial logistic regression model
Source: eBioMedicine. 2024 Mar 26;102:105085. doi: 10.1016/j.ebiom.2024.105085 (PMC10987885; doi:10.1016/j.ebiom.2024.105085)
Supplement: Supplementary [file mmc1.docx]

**Supplementary Material**

**Figure S1**


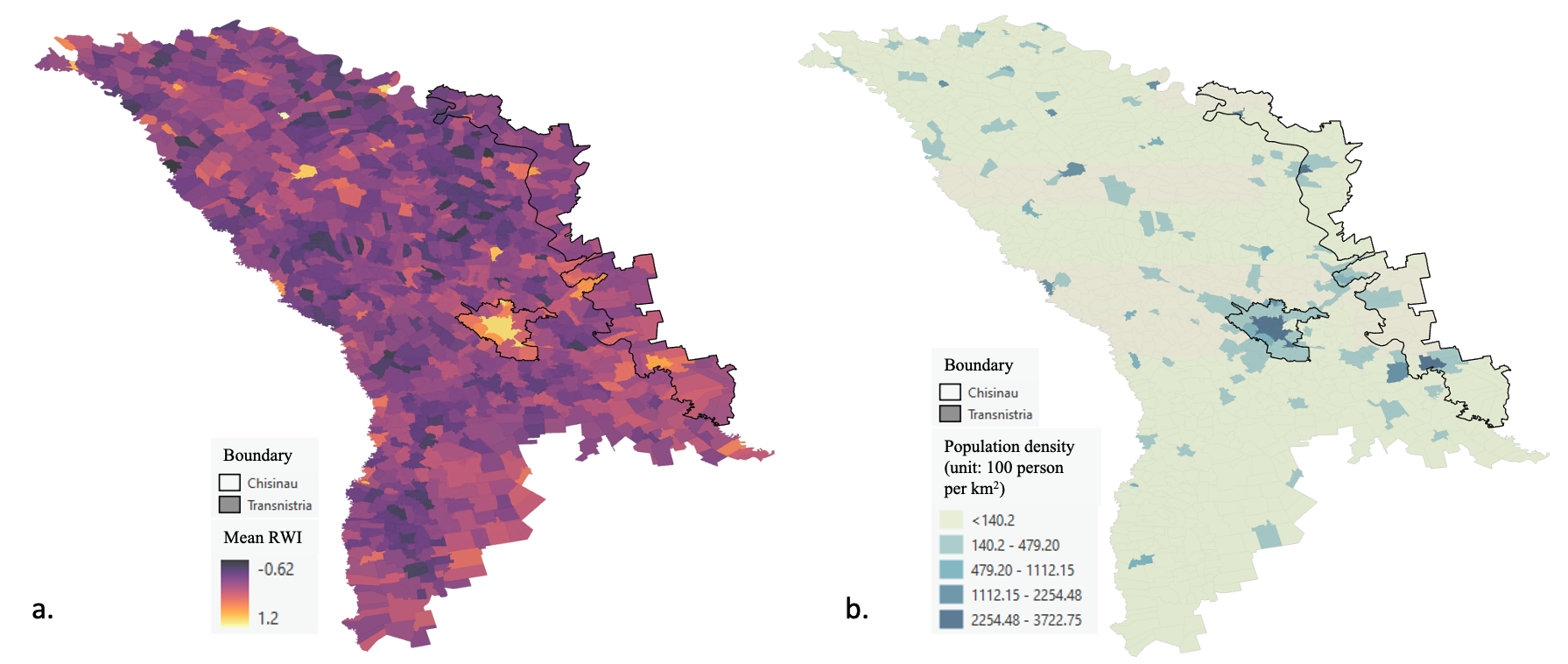


**Figure S1: Spatial distribution of locality-level factors in Moldova. a) Mean of relative wealth index (poverty index); b) Population density (unit: 100 persons per km^2^).**

**Figure S2**


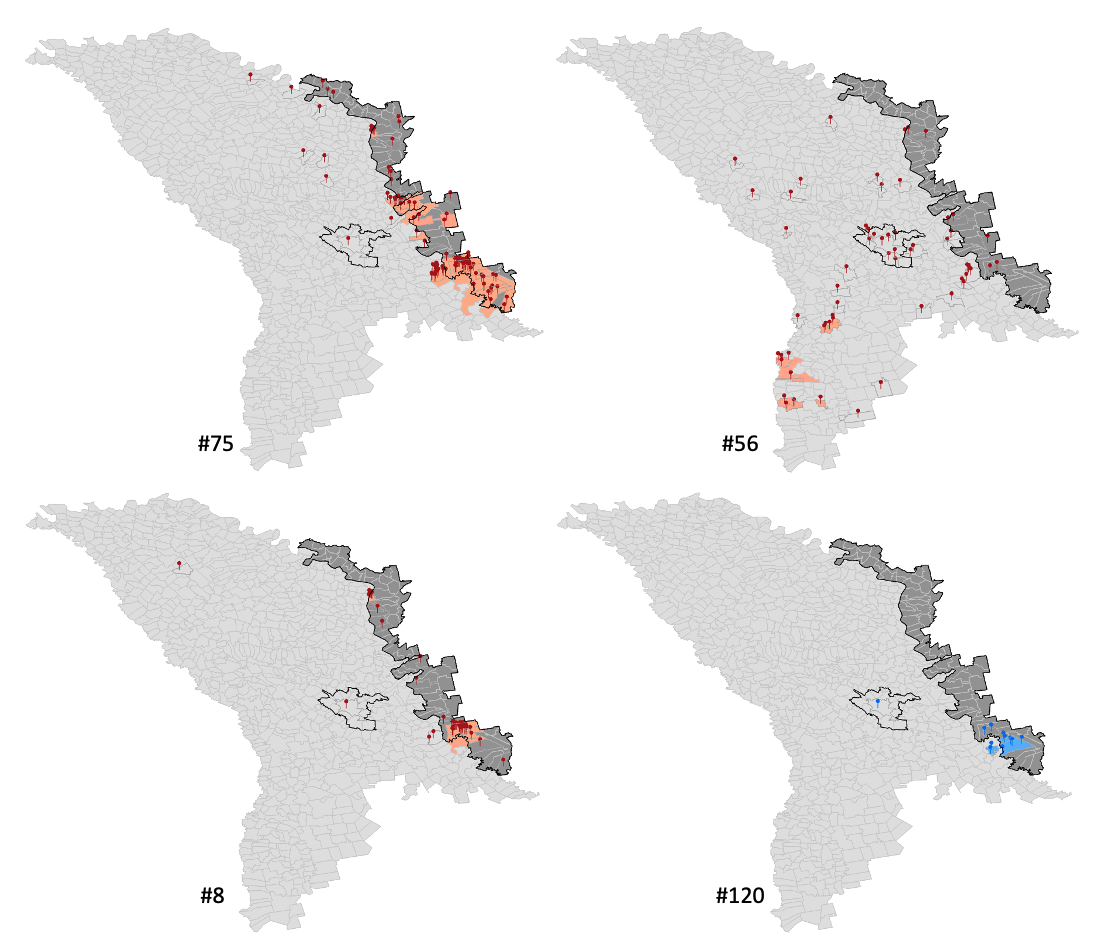


**Figure S2: Sensitivity analysis results: localities in which specific strains of *M. tuberculosis* is focused.**

**Identification of transmission foci using an alternate distance-based mapping approach**

We used an alternative approach to identify transmission foci of the 35 putative *M. tuberculosis* transmission networks in Moldova to test the robustness of our findings presented in the main analysis. We applied a non-parametric distance-based mapping (DBM) using *hotspotr* in R to all culture-positive TB cases.^1^ To produce this model, we denote individuals belonging to one of 35 transmission networks as “cases”, while all other individuals were denoted as “controls”; this approach is then repeated for each of the remaining 34 transmission networks. The DBM method generates 100*100 grid cells covering the all individuals in the study, and each grid is assigned a score from 0 to 1. The score indicates the risk that a cell has more cases (of each specific transmission type) than expected under the null distribution. As our study aggregated cases into a granular/village level, we set the width of smoothing window as a relatively small number (i.e., 0.01) for the DBM analysis prevent over-smoothing. In Figure S1, we used a threshold of 0.95 for visualization; accordingly, the map shows cells scoring above the 95^th^ percentile. The results are consistent with the analysis presented in the main text: transmission networks of Beijing lineage have foci throughout Transnistria and in Chisinau (Figure S1 panel a) while those of the Ural lineage have foci in southern Transnistria and Chisinau (Figure S1 panel b). We note that the DBM method and other similar distance-based methods (such as kernel density estimation) require assigning parameters (e.g., the width of smoothing window in this case) and thresholds for visualization.


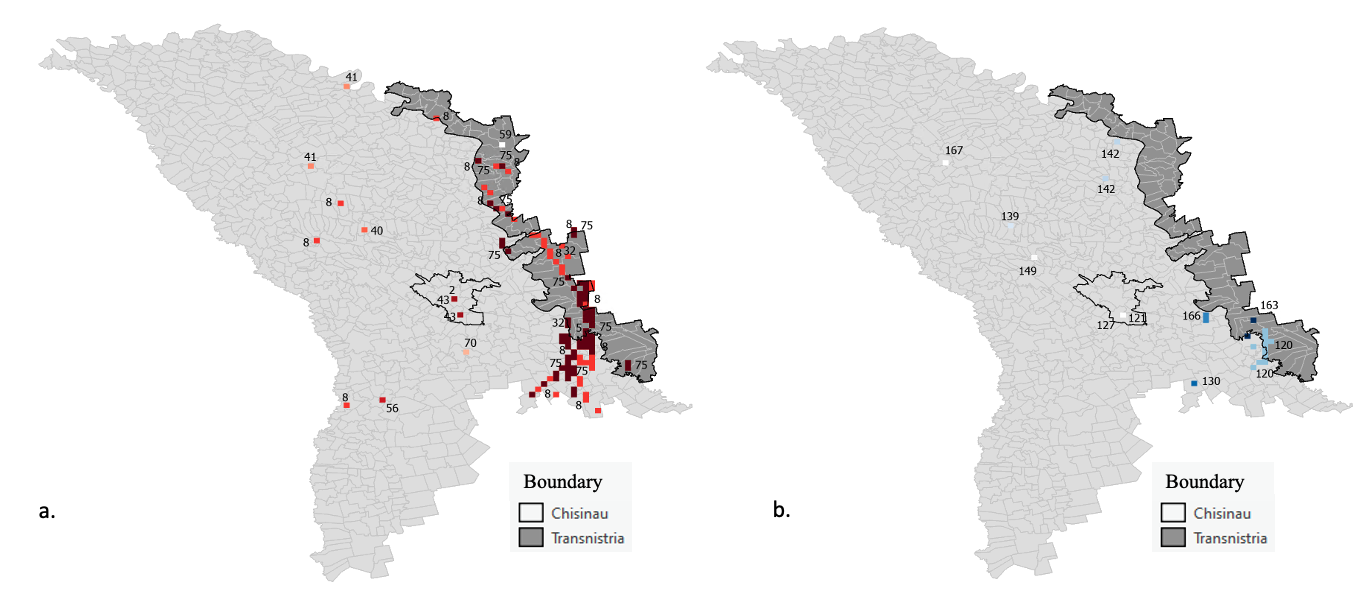


**Figure S3: Regions in which specific strains of *M. tuberculosis* are focused based a non-parametric distance-based measure (DBM). a) Lineage 2.2.1/Beijing transmission foci; b) Lineage 4.2.1/Ural transmission foci.**

**References**

1. Jeffery C, Ozonoff A, White LF, Pagano M. Distance-based mapping of disease risk. *The International Journal of Biostatistics* 2013; **9**(2): 265-90.
